# 14-3-3 Proteins Are on the Crossroads of Cancer, Aging, and Age-Related Neurodegenerative Disease

**DOI:** 10.3390/ijms20143518

**Published:** 2019-07-18

**Authors:** Xiaolan Fan, Lang Cui, Yao Zeng, Wenhao Song, Uma Gaur, Mingyao Yang

**Affiliations:** 1 Institute of Animal Genetics and Breeding, Sichuan Agricultural University, Chengdu 611130, China; 2 Farm Animal Genetic Resources Exploration and Innovation Key Laboratory of Sichuan Province, Sichuan Agricultural University, Chengdu 611130, China

**Keywords:** 14-3-3 proteins, cancer, aging, neurodegenerative diseases, adaptor, chaperone-like

## Abstract

14-3-3 proteins are a family of conserved regulatory adaptor molecules which are expressed in all eukaryotic cells. These proteins participate in a variety of intracellular processes by recognizing specific phosphorylation motifs and interacting with hundreds of target proteins. Also, 14-3-3 proteins act as molecular chaperones, preventing the aggregation of unfolded proteins under conditions of cellular stress. Furthermore, 14-3-3 proteins have been shown to have similar expression patterns in tumors, aging, and neurodegenerative diseases. Therefore, we put forward the idea that the adaptor activity and chaperone-like activity of 14-3-3 proteins might play a substantial role in the above-mentioned conditions. Interestingly, 14-3-3 proteins are considered to be standing at the crossroads of cancer, aging, and age-related neurodegenerative diseases. There are great possibilities to improve the above-mentioned diseases and conditions through intervention in the activity of the 14-3-3 protein family.

## 1. Introduction

Aging is followed by a gradual decline in the functions of multiple organ systems and an increase in the incidence of chronic diseases such as cancer [1], Type 2 diabetes, and Alzheimer’s disease (AD) [2]. Aging is also associated with considerable alterations in internal homeostasis, especially in the immune and endocrine systems, which play a significant role in cancer control. Therefore, aging and carcinogenesis are coupled to each other at the molecular level [3]. By analyzing the transcriptomic data set covering 531 samples at five different time points of aging, Peer et al. found that the aging-associated changes in transcriptomic expression and the transformation characteristics of chronic degenerative diseases (cardiovascular, metabolic, and neurodegenerative diseases) are related to each other and are different from the gene expression characteristics associated with cancer [4]. It seems that aging, chronic degenerative diseases, and tumors affect the human body via different directions. Interestingly, aging and cancer also share similar expression characteristics, for example, the genomic stability during aging and cancer follows a similar pattern at the transcriptome level [5,6] and therapeutic interventions for one of these might allow dual benefits of anti-aging as well as cancer prevention. These strategies include, but are not limited to, caloric restriction [7], drug senolytics [8,9], and so on. Long-term caloric restriction has been shown to delay the development of aging-related diseases in rodents and primates, including cancer [10].

The 14-3-3 protein family is constituted by 28–33 kDa acidic proteins found in all eukaryotes [11]. The 14-3-3 proteins are phosphorylated serine/threonine binding proteins that bind to a variety of kinases, phosphatases, transmembrane receptors, and transcription factors. Hundreds of 14-3-3 ligands have been reported in the human proteome [12,13]. The 14-3-3 proteins are widely expressed, especially in the central nervous system (CNS), and plays a key role in development [14] and disease progression [15]. Playing a similar role as other domains in signaling networks, 14-3-3 proteins generally interact with proteins that are involved in one of the three major functions, i.e., regulation, localization, or catalysis. It is widely accepted that 14-3-3 proteins act in two ways: By acting as adaptors [16] and by displaying chaperone-like activity [17]. By interaction with its partners, 14-3-3 proteins regulate critical biological processes, such as cell proliferation, growth, and apoptosis [18,19]. In addition, 14-3-3 proteins are also involved in the regulation of various tumors [20], metabolic diseases [21], and neurodegenerative diseases [22,23]. The gist of this review article is that 14-3-3 proteins are consistently up- and down-regulated in tumors, aging, and neurodegenerative disease. This suggests that targeting 14-3-3 proteins with specific drug compounds may facilitate a common therapeutic approach against aging, neurodegenerative disease, and cancer.

## 2. The Structure of 14-3-3 Proteins

14-3-3 proteins are present in almost all eukaryotic cells [24]. There are seven human 14-3-3 members according to the amino acid sequences (Table 1), while two isoforms in yeast and up to 13 isoforms in plants have been observed [25].

It is well-accepted that most isoforms of the 14-3-3 proteins can form and function as both homodimers and heterodimers, with an exception of the 14-3-3σ isoform, which preferentially forms homodimers [56,57]. The crystal structures of all seven mammalian 14-3-3 isoforms are available, showing that homodimers or heterodimers of 14-3-3 proteins generally consist of 9 α-helices. Each monomer consists of a bundle of nine antiparallel helices (H1-H9) [58]. The 14-3-3 dimers form cup-shaped structures, with a large, negatively charged, central passage with a diameter of about 35 Å, a width of 35 Å, and a depth of 20 Å, containing two ligand-binding grooves [59,60]. These grooves include the side chains of Lys49, Arg56, Arg127, and Tyr128 (residue numbering corresponds to the isoform of 14-3-3). The monomeric subunits form a dimer through their N-terminal helices and the linkage of the salt bridge connects the dimer between the first two helices of one monomer and the fourth helix of the other monomer [61]. The dimers bind the target proteins by three consensus phosphopeptide sequences: Motif I (RSXpSXP), motif II (RX(Y/F)XpSXP), and motif III (pSX1-2–COOH), wherein pS represents a phosphorylated serine/threonine and X is any residue [62,63]. These phosphopeptide binding sites are present in both monomer units of 14-3-3, therefore this protein can bind to both phosphopeptides simultaneously; they can be from the same target protein, or two different target proteins [64]. Furthermore, 14-3-3 proteins serve as adaptors or linkers. Depending upon the phosphorylation state of their specific recognition partners, 14-3-3 proteins bind their targets in order to stabilize the structure, phosphorylate and control their targets at the degradation level [65,66,67], localize and distribute between the different cellular compartments [68], and ultimately modulate their own interactions with other proteins.

However, 14-3-3 proteins also have the chaperone-like activity, i.e. 14-3-3ζ has been reported to dissolve heat-aggregated citrate synthase in vitro and has also been shown to interact with the heat shock proteins (HSP), HSP70/HSP40 chaperone to promote its reactivation [67]. This chaperon-like activity of the 14-3-3 family proteins is very different from the well-characterized phosphorylation-dependent interaction of 14-3-3 with multiple target proteins. Neither the phospho-serine binding groove nor the flexible C-terminal extension have been proven to be necessary for 14-3-3 chaperone activity [69]. Regardless of the use of any model substrate, 14-3-3 monomeric forms generally have higher activity than the dimeric form [70]. Studies by Sluchanko et al. have shown that exposure of the dimer interface may play a role in 14-3-3 proteins’ molecular chaperone mechanism [71,72]. Joanna et al. reported that the N-terminal helices of 14-3-3zeta may also play a role in chaperone action, whereby a D21N mutation may provide the key to the chaperone activity [72]. To summarize, the mechanism of 14-3-3 anti-aggregation activity appears to be similar to the unrelated small heat shock proteins (sHsps) and is independent of ATP. The N-terminal portion of 14-3-3 contains a hydrophobic region and hides the intrinsic barrier that is critical for protein dimerization and appears to be important for the development of unfolded/misfolded proteins. Many different factors that promote dimerization enhance the chaperone-like activity of 14-3-3.

## 3. 14-3-3 Proteins Have Consistent Expression Patterns in Aging and Cancer

### 3.1. Cancer

Much work has been dedicated to understand the role of 14-3-3 proteins in cancer. Because of the lack of significant catalytic activity, the contribution of 14-3-3 proteins to cancer is primarily related to the regulation of oncoproteins and tumor suppressor proteins. The detailed information regarding the regulation of different types of cancers by 14-3-3 protein isoforms has been summarized in Table 1.

As shown in Table 1, the majority of 14-3-3 isoforms are elevated in almost all types of tumors, except the σ isoform, which is down-regulated in some cancer types. In breast, gastric, prostate, lung, and liver cancers, an association is seen with elevated levels of most 14-3-3 isoforms, whereas in leukemia, renal, and glioma cancers, only few specific isoforms have been reported to exhibit abnormal expression. There are a large number of reports on the regulatory mechanisms about the ζ and σ isoforms in tumors (see Table 1), therefore, in the following paragraphs, we will discuss their detailed regulatory mechanism in cancers.

The ζ isoform among the 14-3-3 protein family is the one with most abundant research reports in a multitude of cancers. The 14-3-3ζ isoform is highly expressed in a variety of cancers, including breast, ovarian, prostate, lung, and stomach cancers [26,73]. This high expression of 14-3-3ζ has been associated with (but not limited to) poor prognosis and resistance to these cancers [74]. 14-3-3 promotes survival of cancer cells through either binding to the p85 regulatory subunit of PI3K and activating Akt [75], or inactivating the tumor suppressor genes p53 and p21 [76].

The 14-3-3ζ isoform plays an important role in another important cancer signaling pathway, which is the Wnt5a/ROR1 signal transduction pathway, and promotes the migration and proliferation of chronic lymphocytic leukemia [77]. The 14-3-3ζ isoform likely functions via binding to the FOXO3a transcription factor and facilitating its transport to the cytoplasm, which in turn results in the enhanced proliferation of tongue cancer cells. In breast cancer cells, 14-3-3ζ brings forth contextual changes of Smad partners from p53 to Gli2 and therefore facilitates the switch from the tumor suppressive function of TGFβ to its metastasis-promoting activity [78].

The 14-3-3σ isoform attracts particular attention, which is considered to be a tumor suppressor protein whose down-regulation has been frequently detected in tumor specimens of many types of cancer. Also, 14-3-3σ was found to be a potent tumor suppressor involved in ErbB2-driven breast cancer initiation and metastasis [79]. There is evidence that correlates the low expression of 14-3-3σ to hypermethylation of the 14-3-3σ promoter, leading to gene silencing [80,81]. The promoter regions of 14-3-3σ gene displayed abnormal methylation in breast, lung, liver, ovarian, bladder, and prostate cancers [82,83,84,85,86]. Therefore, 14-3-3σ methylation can be used as a diagnostic indicator for these tumors [32]. 14-3-3σ has also been shown to be involved in the regulation of the energy metabolism of cancer cells. 14-3-3σ targets c-Myc for ubiquitination and proteasome-mediated degradation to suppress tumor metabolic reprogramming [87]. Recently, it has been shown that the 14-3-3ζ and 14-3-3σ isoforms play an opposite role in the regulation of tumor suppressor or metastasis-promoting functions of transforming growth factor beta (TGFβ) signaling during cancer [78,88]. In short, the 14-3-3 proteins in cancers mostly work as adaptors to bind their phosphorylated target proteins to regulate the occurrence, development, metastasis, and invasion of tumors.

### 3.2. Age-Related Neurodegenerative Disease

The 14-3-3 proteins exhibit chaperone-like activity, wherein they contain a nuclear localization sequence (NLS) through which they can transport target proteins to the nucleus [25]. The pathogenesis of certain neurological diseases, such as Alzheimer’s disease (AD), Parkinson’s disease (PD), amyotrophic lateral sclerosis (ALS), schizophrenia, and bipolar disorder involve misfolding and excessive aggregation of proteins. Because of their chaperone-like activity, 14-3-3 proteins may play a role in these disease states [89,90,91]. In fact, 14-3-3 proteins are highly expressed in the brain, accounting for about 1% of the total amount of soluble brain proteins [89]. Also, the 14-3-3 isoform-specific functional knock-out mice have shown some syndrome phenotypes. Multiple studies in 14-3-3 isoform-specific K/O mouse models, as summarized in Table 2, have been very helpful in understanding 14-3-3 isoform-specific functions in the brain. Looking at the chaperone-like activity of 14-3-3 proteins, it comes as no surprise to see their involvement in a number of neurological disorders.

**Figure 1:**
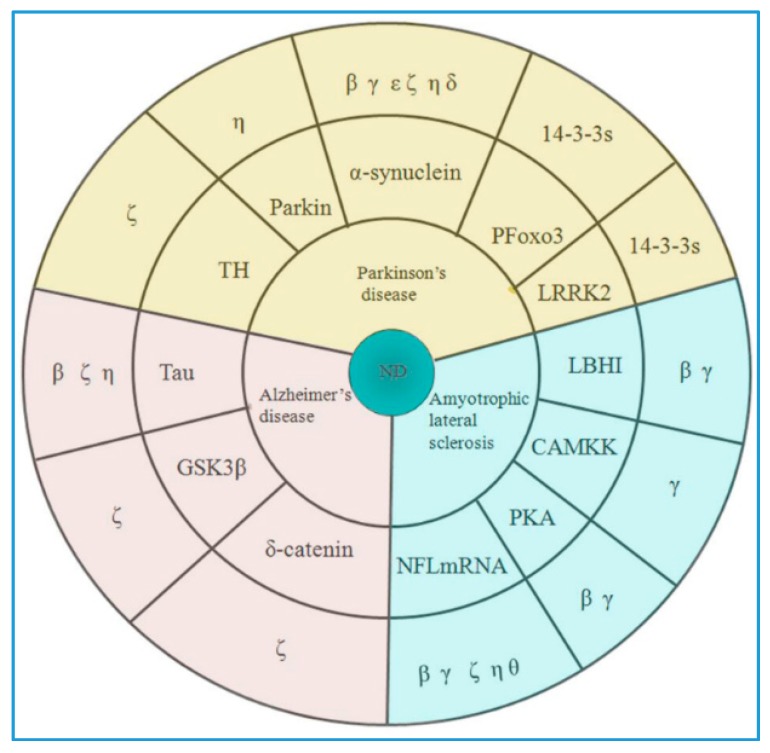
The 14-3-3 isoforms in neurodegeneration. ND: Neurodegenerative disease; TH: Tyrosine hydroxylase; LRRK2: Leucine-rich repeat kinase 2; LBHI: lewy body-like hyalineinclusions; CAMKK: Calcium/calmodulin-dependent protein kinase kinase; PKA: Protein kinase A.

The association of 14-3-3 proteins with neurodegenerative diseases is further strengthened by their presence in Lewy bodies (LBs) and neurofibrillary tangles (NFTs) of AD brain sections. Several 14-3-3 isoforms are able to interact with specific proteins involved either in PD, ASL, or AD (Figure 1).

#### 3.2.1. Parkinson’s Disease

Most of the 14-3-3 proteins are capable of interacting with α-synuclein, which is a regulator of the mitogen-activated protein kinase (MAPK) pathway, and therefore play an important role in the synthesis of dopamine [101]. Connotations between 14-3-3 (β and ε isoforms) and α-synuclein occurs either in cytosolic or membrane fractions of rat brain homogenate [102]. In fact, 14-3-3 and α-synuclein can be obtained by co-immunoprecipitation in the mammalian brain [103]. The 14-3-3η isoform strongly affect the products and the kinetics of α-synuclein aggregation in vitro by binding to α-synuclein oligomers. Overexpression of the 14-3-3η isoform results in reduced α-synuclein toxicity in cellular models [104]. One possible mechanism for this could be that the 14-3-3 protein is sequestered by the interaction with α-synuclein, resulting in a loss of 14-3-3 function, which is involved in the pathogenesis of PD.

Besides the α-synuclein, the 14-3-3ζ isoform also binds and stimulates the activation of tyrosine hydroxylase (TH), the rate-limiting enzyme in the biosynthesis of catecholamine [105]. The 14-3-3η isoform interacts with parkin, which is an ubiquitin E3 ligase, leading to protein degradation. The 14-3-3η–parkin association leads to the suppression of ubiquitin-ligase activity of parkin, which is one of the causes of PD [106].

LRRK2 and phosphorylated FOXO3a are also the interacting partners of 14-3-3 proteins [107]. FOXO3a localizes in LBs and recently a hypothesis was proposed suggesting the formation of a complex, including FOXO3a, α-synuclein, and 14-3-3 proteins, which promotes cell survival [108].

#### 3.2.2. Alzheimer’s Disease

Tau is a major microtubule-associated protein in neurons, which can bind and stabilize microtubules. Tau phosphorylation reduces its affinity for microtubules and it is reported that tau is hyperphosphorylated in AD [109]. 14-3-3 proteins have been detected in NFT of AD patients, with 14-3-3ζ being the most immuno-reactive [110,111]. Further study has demonstrated that 14-3-3ζ facilitates GSK3β-dependent phosphorylation of tau by enhancing the affinity of GSK3β for tau [112]. The 14-3-3ζ isoform also binds to δ-catenin [113], a brain protein first discovered in the interaction with presenilin 1 [114].

In addition to the evidence of specific interactions with proteins associated to neurodegenerative diseases, 14-3-3 proteins also exhibit protective effects on dopaminergic neurons [115]. Indeed, 14-3-3θ, γ, and ε isoforms reduce the cellular toxicity induced by neurotoxins, causing cell death in dopaminergic cells [23,116]. It has also been suggested that 14-3-3 proteins may be involved in the chelation and degradation of toxic oligomers and aggregates by promoting the formation of aggresomes [117]. Recently, 14-3-3 proteins recognized phosphorylated transcription factor EB (TFEB) and affected the autophagy, which is strongly correlated with neurodegenerative disease [118]. Thus, looking at the functions of 14-3-3 proteins in age-related neurodegenerative disease, the potential development of drugs to therapeutically target 14-3-3 protein-protein interactions (PPIs) could be a good approach for the treatment of these kinds of diseases.

## 4. Aging Process

The 14-3-3 proteins are shown to be involved in many metabolic and autophagy regulatory pathways, such as Insulin/insulin-like growth factor signalling (IIS), AMP-activated protein kinase (AMPK), mechanistic target of rapamycin (mTOR) and MAPK, and these pathways play direct roles in the aging process. Thus, the 14-3-3 protein family may play a role in regulation of aging. The first study to explore the involvement of the 14-3-3 protein family on the lifespan regulation was carried out on *C. elegans*. Wang et al. found that in lifespan regulation, 14-3-3 proteins were co-expressed with DAF-16 and SIR-2.1 and DAF-16/ Forkhead box O (FOXO) interacted physically with 14-3-3 proteins [119], suggesting that in *C. elegans,* the 14-3-3 protein regulate the lifespan by synergy with SIR-2.1 and DAF-16/FOXO. In another report, Berdichevsky et al. demonstrated that SIR-2.1 and 14-3-3 activated DAF16 and extended the life span in a stress-dependent pathway in *C. elegans* [120]. Also, 14-3-3 proteins promoted the life span by both FOXO/daf16-dependent and independent manners [121]. To sum up, the role of 14-3-3 proteins in lifespan regulation in *C. elegans* is mostly by interacting with FOXO/DAF16.

In *Drosophila*, there are two isoforms of proteins, ε and ζ. Nielsen and colleagues found that the mutations in 14-3-3ε resulted in increased stress-induced apoptosis, growth inhibition, and prolonged lifespan, which were associated with increased FOXO activity [122]. Both 14-3-3 protein isoforms regulated two interacting components of mTOR signaling in *Drosophila* and regulated the translation of tumor protein (Tctp) and Rheb GTPase during organ growth [123]. It is already well-known that FOXO and TOR are two proteins that participate extensively in the aging process [124,125]. Therefore, it can be stated that 14-3-3 proteins participate in the lifespan by regulating the activity of these longevity proteins in *Drosophila*.

The 14-3-3 proteins have also been reported to participate in the process of metabolic diseases, such as obesity and diabetes [126]. The 14-3-3 protein interaction partner, heart-isomerized phospho-fructose-2-kinase/fructose-2,6-bisphosphatase (PFK-2), is involved in gluconeogenesis and glycolysis [127]. 14-3-3β and 14-3-3γ have been reported to participate in human PPARγ2 transactivation and hepatic lipid metabolism [128]. 14-3-3ζ and 14-3-3γ have been reported to be elevated in visceral and subcutaneous adipose tissue of obese individuals [129]. 14-3-3ζ-overexpressing mice had significantly higher body weights and fat masses when fed a high fat diet [130]. Significant changes in RNA and protein levels of 14-3-3ζ, ε, θ, and η in a murine model of Type 1 diabetes mellitus (T1DM) were detected [131]. Thus, it can be put forth that 14-3-3 proteins contribute to the development of metabolic diseases.

In *Saccharomyces cerevisiae*, upon deleting the 14-3-3 protein, Bmh1 increased the stress response and prolonged the lifespan [132]. The isoform β negatively regulated the glioblastoma cells senescence via the ERK-SKP2-p27 pathway [133]. The 14-3-3η protein and the downstream MAPK were thought to be effective in age-related cardiac dysfunction [134]. Network analyses have shown that skin aging triggered significant downregulation of 14-3-3 sigma [135]. Therefore, it can be firmly stated that 14-3-3 proteins play a very important regulatory role during aging.

## 5. Conclusions and Challenges

**Figure 2:**
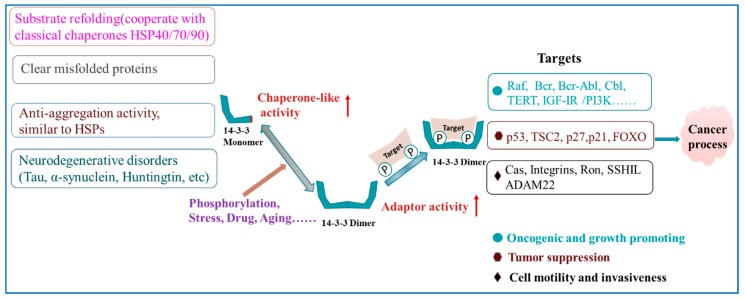
Schematic showing the action mechanisms of 14-3-3 proteins. The right half of the scheme shows the phosphopeptide-binding adaptor function of 14-3-3 proteins and the left half describes the chaperone-like function. The function of the phosphopeptide-binding adapter is primarily contributed by the dimeric form of 14-3-3, while the chaperone-like activity is primarily attributed to their monomeric form. Various factors (phosphorylation, stress, drug, aging, etc.) can disturb the balance between the dimeric and monomeric forms of the 14-3-3 proteins. Further details can be seen in the text. IGF-I: Insulin and insulin related growth factor I; TERT: Telomerase reverse transcriptase; Ron: Recepteur d’Origine nantais; SSH1L: Cofilin-phosphatase slingshot-1L; ADAM22: A disintegrin and metalloprotease 22.

The 14-3-3 protein family plays a major role in aging, cancer, and aging-related neurodegenerative disease. During these disease states, the majority of the 14-3-3 proteins are up-regulated. This means a careful reduction of 14-3-3 activity in these processes or diseases may be beneficial in alleviating the relevant phenotype. However, the activity of 14-3-3 in regulating tumors and neurological diseases is slightly different (Figure 2). In tumors, 14-3-3 proteins play the role of adaptors by regulating the phosphorylation of the target sites in order to regulate protein activity, proliferation, apoptosis, metastasis, and survival of tumor cells. All of this regulation is predominantly based on the presence of a special phosphopeptide-binding amphipathic groove and on the dimeric status of 14-3-3.

In neurological diseases, 14-3-3 proteins mostly exhibit chaperone-like activities to interact with the protein aggregates. This activity of 14-3-3 proteins prevent the aggregation of partially folded or misfolded proteins or pro-proteins, thereby protecting cells from the accumulation of potentially harmful oligomers of unfolded protein intermediates. Therefore, it represents an integral part of the overall cyto-protection system and this protection is mostly associated with the monomeric forms. Various factors (stress, drugs, aging, etc.; see Figure 2) can alter the balance between 14-3-3 protein dimers and their monomeric forms, thereby affecting their mode of action.

The members of the 14-3-3 protein family can bind hundreds of target proteins and perform essential roles in human development, health, and pathological processes. However, many challenges still exist around this protein family. As 14-3-3 proteins function as dimers and monomers, the 14-3-3 isoforms may have functional redundancy. Alterations in the specific isoform levels may thus have an indirect effect by changing the balance of the 14-3-3 proteins. Furthermore, due to the similarity of the 14-3-3 family protein structure, it is very challenging to specifically inhibit one isoform’s function. Strategies to effectively regulate 14-3-3 activity need to be developed in order to utilize them as therapeutic candidates.

## Figures and Tables

**Table 1:** 14-3-3 protein isoforms in cancers.

14-3-3 Isoform	Cancer Type	Expression	Reference
14-3-3ζ	Breast, lung, pancreas, esophageal, head and neck, oral, colon, chronic myeloid leukemia, ovarian	**↑**	[26,27,28,29,30,31]
14-3-3σ	Lung, breast, esophageal, chronic myeloid leukemia, uterine, ovarian, skin	**↓**	[32,33,34,35,36]
	Liver, pancreatic ductal	**↑**	[37,38]
14-3-3β	Lung, astrocytoma, glioma, colorectal, gastric squamous, liver	**↑**	[39,40,41,42]
14-3-3ε	Renal, liver, squamous, breast, gastric	**↑**	[43,44,45,46]
14-3-3γ	Liver, breast, lung	**↑**	[47,48,49]
14-3-3η	Liver, prostate, squamous, glioma	**↑**	[49,50,51,52]
14-3-3τ/θ	Breast, lung, glioma, prostate	**↑**	[53,54,55]

Red ****↑**** = elevated expression; blue **↓** = decreased expression.

**Table 2:** 14-3-3 isoform knock-out phenotypes in mice.

K/O Isoform	Phenotypes	References
14-3-3ζ	Schizophrenia, autism spectrum disorder, and bipolar disorder; reduced learning, memory, and prepulse inhibition and locomotor hyperactivity	[92,93]
14-3-3ε	Schizophrenic behavior; increased locomotor activity and sociability and decreased working memory	[94,95]
14-3-3γ	Hyperactive and depressive-like behavior; sensitive responses to acute stress	[96]
14-3-3ζ and 14-3-3ε	Neuronal migration and pigmentation defects and neural progenitor cells	[14,97,98]
14-3-3 functional knock-out	Schizophrenic behavior; synaptic alterations	[99,100]
